# Transcriptome Analysis of the Mud Crab (*Scylla paramamosain*) by 454 Deep Sequencing: Assembly, Annotation, and Marker Discovery

**DOI:** 10.1371/journal.pone.0102668

**Published:** 2014-07-23

**Authors:** Hongyu Ma, Chunyan Ma, Shujuan Li, Wei Jiang, Xincang Li, Yuexing Liu, Lingbo Ma

**Affiliations:** 1 East China Sea Fisheries Research Institute, Chinese Academy of Fishery Sciences, Shanghai, China; 2 Key Laboratory of East China Sea and Oceanic Fishery Resources Exploitation, Ministry of Agriculture, Shanghai, China; University of North Carolina at Charlotte, United States of America

## Abstract

In this study, we reported the characterization of the first transcriptome of the mud crab (*Scylla paramamosain*). Pooled cDNAs of four tissue types from twelve wild individuals were sequenced using the Roche 454 FLX platform. Analysis performed included *de novo* assembly of transcriptome sequences, functional annotation, and molecular marker discovery. A total of 1,314,101 high quality reads with an average length of 411 bp were generated by 454 sequencing on a mixed cDNA library. *De novo* assembly of these 1,314,101 reads produced 76,778 contigs (consisting of 818,154 reads) with 5.4-fold average sequencing coverage. The remaining 495,947 reads were singletons. A total of 78,268 unigenes were identified based on sequence similarity with known proteins (E≤0.00001) in UniProt and non-redundant protein databases. Meanwhile, 44,433 sequences were identified (E≤0.00001) using a BLASTN search against the NCBI nucleotide database. Gene Ontology (GO) analysis indicated that biosynthetic process, cell part, and ion binding were the most abundant terms in biological process, cellular component, and molecular function categories, respectively. Kyoto Encyclopedia of Genes and Genome (KEGG) pathway analysis revealed that 4,878 unigenes distributed in 281 different pathways. In addition, 19,011 microsatellites and 37,063 potential single nucleotide polymorphisms were detected from the transcriptome of *S. paramamosain*. Finally, thirty polymorphic microsatellite markers were developed and used to assess genetic diversity of a wild population of *S. paramamosain*. So far, existing sequence resources for *S. paramamosain* are extremely limited. The present study provides a characterization of transcriptome from multiple tissues and individuals, as well as an assessment of genetic diversity of a wild population. These sequence resources will facilitate the investigation of population genetic diversity, the development of genetic maps, and the conduct of molecular marker-assisted breeding in *S. paramamosain* and related crab species.

## Introduction

The mud crab (*Scylla paramamosain*), a large marine portunid species, is widely distributed along the southeast China coasts and other Asian countries, such as Japan, Vietnam, and the Philippines. *S. paramamosain* is one of the most important aquaculture animals and marine fishery resources in China because of its large market demand, wonderful flavor, fast growth rate, large size, and high commercial value. Records of *S. paramamosain* aquaculture date back to over 100 years in China [1] and more than 30 years in other Asian countries [2]. In recent years, the aquaculture production has stably reached 110,000 tons per year in China [3]. However, the yield per unit is still at a low level, which can be attributed to various problems, such as growth and development, nutrition, diseases, as well as administration patterns. Meanwhile, the wild resources of *S. paramamosain* are rapidly decreasing because of seawater pollution, over-fishing, and habitat environment changes.

Population genetic diversity, differentiation, and phylogenetic relationship of *S. paramamosain* have been well investigated [4]–[9]. Functional genes and their effects on growth, development, and disease-resistance have also been studied in recent years, in order to seek a new way for increasing aquaculture yield and boosting aquaculture industry. These characterized functional genes included crustin [10], scygonadin [11], vasa [12], SpMyD88 [13], HSP70 [14], and so on. However, the regulatory mechanism of functional genes to growth, development, and disease resistance of *S. paramamosain* are still poorly understood. Genetic markers have been identified [15]–[18] for construction of genetic maps, quantitative trait locus (QTL) mapping, and molecular marker-assisted selection [19]. Nevertheless, the available molecular markers are extremely limited for conducting the above-mentioned works in *S. paramamosain*.

Sufficient genome or transcriptome resources would be useful for intensive study on gene expression and variation, molecular marker application, and genome comparison. At present, only 800 genes (or partial genes) and 370 microsatellite sequences can be obtained from the NCBI GenBank database as of July 25, 2013. Small-scale cDNA sequencing was performed in *S. paramamosain*, which produced 3,837 unique sequences and 411 microsatellite repeat motifs [20]. To our knowledge, neither whole genome sequencing project, nor large-scale next-generation sequencing research of *S. paramamosain* was reported to date.

Next-generation sequencing techniques, such as 454 sequencing [21] have shown great potential in producing large-scale functional genes and molecular markers at the genome level, especially in non-model organisms. In recent years, the 454 sequencing technique has been broadly applied in many species, such as corals (*Acropora millepora*) [22], pines (*Pinus* spp.) [23], orange-spotted grouper (*Epinephelus coioides*) [24], yesso scallops (*Patinopecten yessoensis*) [25], and Adriatic sturgeon (*Acipenser naccarii*) [26]. However, no 454 sequencing data is available in *S. paramamosain* so far. The lack of enough genome sequences has seriously hindered studies on gene regulatory mechanism, population genetic structure, and molecular marker-assisted selection.

In this study, we first constructed a mixed sequencing library using multiple kinds of tissues and several individuals. Next, we conducted 454 sequencing, de novo assembly, and gene annotation. Furthermore, we discovered a large number of microsatellites and single nucleotide polymorphisms (SNPs). Finally, we developed a set of polymorphic microsatellites and estimated genetic diversity of a wild *S. paramamosain* population. The findings in this study can be helpful in gene function demonstration, population genetic diversity investigation, genetic maps construction, and molecular marker-assisted selection in *S. paramamosain* and related crab species.

## Materials and Methods

### Ethics Statement

All animal experiments in this study were conducted in accordance with relevant national and international guidelines. Our project was approved by East China Sea Fisheries Research Institute. In China, catching wild mud crab from seawater does not require specific permits. Our study does not involve endangered or protected species.

### Sampling of experimental animals

A total of 12 wild individuals of *S. paramamosain* were collected from the coast (22° 44′ N 113° 85′ E) of Shenzhen City, Guangdong Province, China. Their body weights ranged from 151 to 203 g. The wild crabs were temporarily reared in laboratory for three days. Four kinds of tissues, including muscle, hepatopancreas, eyestalk, and blood, were immediately collected from each sample, rapidly flash-frozen in liquid nitrogen, and stored at −80°C in a refrigerator. In addition, a wild population with 32 individuals was collected from Qinglan Port (19° 47′ N 110° 85′ E) in Wenchang City, Hainan Province, China. These 32 subjects were used to estimate microsatellite variations.

### Nucleic acid extraction and cDNA synthesis

Genomic DNA was extracted from muscle tissue of the 32 wild individuals using the traditional phenol-chloroform extraction method as described in the literature [27]. DNA quality was measured with 1.5% agarose gel electrophoresis. DNA concentration was assessed using a spectrophotometer and adjusted to 50 ng/µl. Finally, genomic DNA was stored at −20°C in a refrigerator until further use.

Total RNAs were extracted from four kinds of tissues using TRIzol Reagent (Invitrogen, USA) according to the protocol of the manufacturer. The quality and concentration of total RNAs were assessed using a GeneQuant Pro spectrophotometer (Pharmacia Biotech Limited, UK) and agarose gel electrophoresis. Total RNAs from each tissue were pooled and then purified to get mRNA using the Oligotex mRNA Kit (Qiagen, Germany) following the protocol of the manufacturer. The quality and concentration of mRNA were measured using a GeneQuant *Pro* spectrophotometer. cDNA was synthesized using SMART PCR cDNA Synthesis Kit (Clontech, US) according to the manufacturer’s instruction. In this process, a 3′ terminal PCR primer (5′ – ATTCTAGAGGCCGAGGCGTGCAG (dT_18_) VN–3′) with *Bsg* I recognition site was introduced. Double-stranded cDNA was synthesized and then purified. Restriction enzyme *Bsg* I (NEB, US) was used to remove the poly A tail of the cDNA. In this study, cDNA was not normalized so as to obtain as many low-expressed transcripts as possible.

### Library construction and 454 sequencing

A 454 library was constructed using the Roche GS FLX Titanium Rapid Library Preparation Kit according to the manufacturer’s instruction. The library was sequenced using a GS FLX Titanium Sequencing Kit XLR70 on Roche 454 Genome Sequencer FLX Titanium (Roche, Switzerland). In this study, 3.6 million beads were sequenced in a total of one run reaction, of which 0.8 million were sequenced in the former quarter run, another 0.8 million in the later quarter run, and 2 million in the final half run. The raw 454 data were processed by filtering the weak signals and low-quality reads, and trimming primers and adaptors, in order to obtain high quality reads. Finally, all high quality reads were deposited in the NCBI Short Read Archive (SRA) database with the accession numbers SRR1310331∼SRR1310333.

### Sequence assembly and functional annotation

To obtain unique transcripts, all high quality reads were assembled by de novo assembly using the MIRA 3.2.0 software [28] with default assembly parameters. The association between the length of contig and the number of reads assembled into a contig was assessed using the SPSS software version 11.5 (http://www-01.ibm.com/software/analytics/spss/). Contigs and singletons were clustered using the CD-HIT software with the parameter of sequence identity at ≥95% [29]. Only one contig or singleton (the longest one) was selected from each cluster and used as a representative. All representatives from different clusters formed the original unigene library. The original unigenes were then compared with the sequences in UniProt and NR protein databases using the BLASTX tool (ftp://ftp.ncbi.nlm.nih.gov/blast/executables/blast+/LATEST/). Only the proteins which have the highest similarity (identity rate≥30% and E≤0.00001) with the query sequences were used for annotation. When several query sequences were annotated to one protein, the longest one was chosen as the final unigene. The query sequences which failed to annotate were treated as final unigenes too. Thus, the final unigene library consisted of contigs and singletons. Moreover, the original unigenes were compared with the sequences in NCBI NT database with an E-value threshold of 0.00001 to search useful information. The unigene sequences and their annotation are provided in File S1 and File S2, respectively.

GO analysis (level 2 and 3) was performed to annotate and classify the final unigenes based on BLASTX results against the Uniprot database. Kyoto Encyclopedia of Genes and Genomes (KEGG) analysis was carried out to classify the unigenes into specific pathways. In this process, the KEGG automatic annotation server was used to complete the KEGG Orthology (KO) and KEGG pathway annotation. The GO and KEGG pathway annotation results are shown in File S2.

### Molecular marker discovery

Microsatellites in unigenes were identified using the MISA software (http://pgrc.ipk-gatersleben.de/misa/) with the following default settings: the minimum repeat number was ten for single-nucleotide, six for di-nucleotide, and five for tri-, tetra-, penta- and hexa-nucleotides. To develop polymorphic microsatellite markers and estimate genetic diversity of a wild population, a total of 78 pairs of primers were designed based on the randomly selected microsatellite sequences using the Primer Premier 5.0 software (http://www.premierbiosoft.com/). These microsatellite loci were genotyped on 32 wild individuals. PCR was conducted as described in the literature [30]. Statistical analysis was performed using the POPGENE 1.31 software [31]. All microsatellites identified in unigenes are listed in File S3. The PCR primer sequence, annealing temperature, and diversity indices are shown in File S4.

Potential SNPs were detected using the SSAHASNP software [32] with default parameters. This process was achieved by aligning all high quality reads to 28,534 unigenes (all of them are contigs, except singletons). The minimum coverage threshold was set as five for SNPs and indels. All potential SNPs detected in unigenes are listed in File S5.

## Results

### 454 sequencing and *de novo* assembly

**Figure 1:**
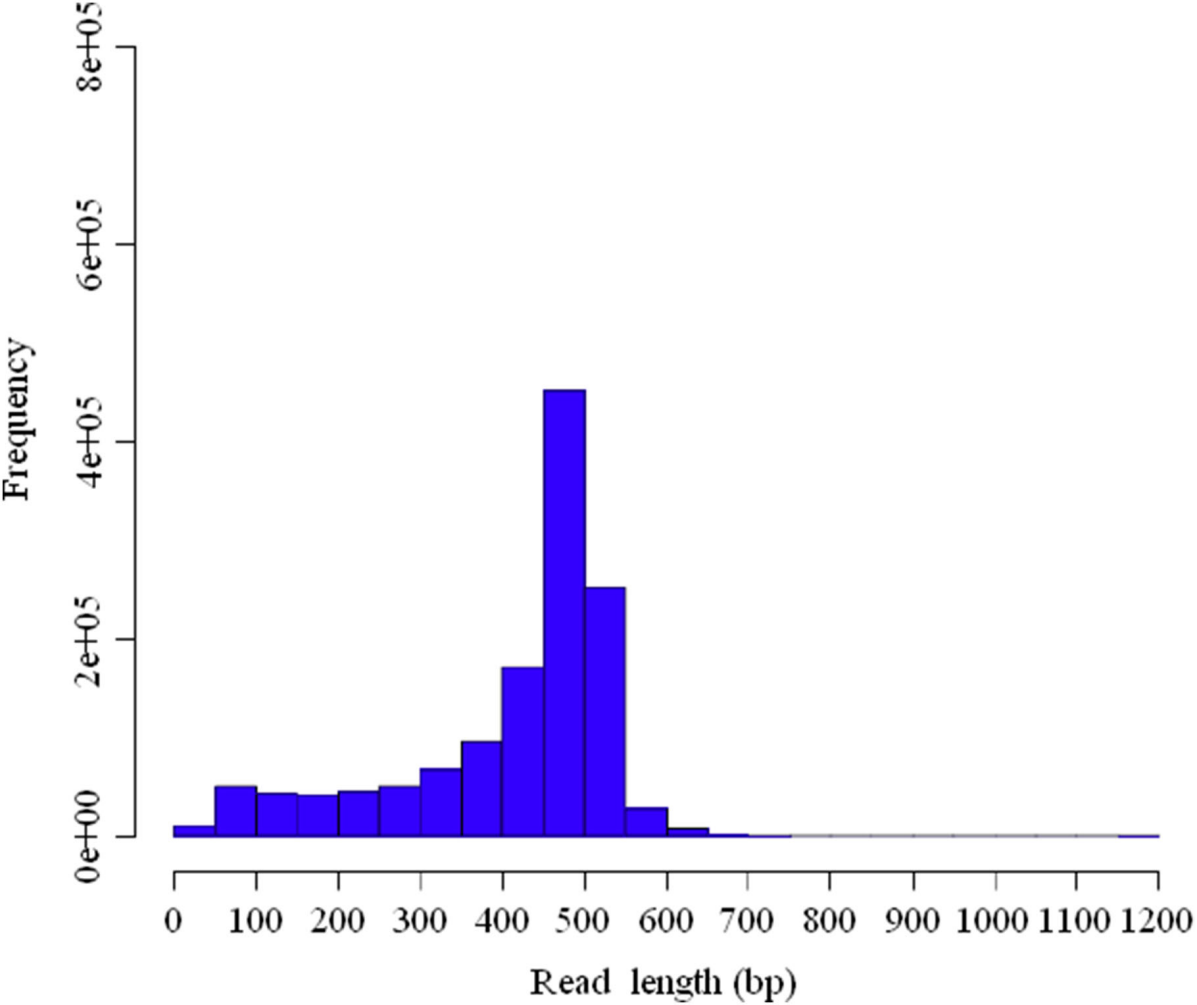
The Size distribution of reads generated from 454 FLX platform pyrosequencing.

In this study, the former quarter run generated 256,228 reads with an average length of 393 bp; the later quarter run produced 331,441 reads with an average length of 420 bp; and the final half run obtained 726,432 reads with an average length of 413 bp (Table 1). A total of 540 Mbp data in size were achieved, which consisted of 1,314,101 high quality reads with an average length of 411 bp. The size distribution of these high quality reads is shown in Figure 1. All high quality reads are available from the NCBI Short Read Archive (SRA) database under the accession numbers SRR1310331∼SRR1310333.

**Table 1:** Characteristics of reads generated from 454 pyrosequencing.

Category	The former quarter run	The later quarter run	The final half run	Total
Raw sequencing reads	398,920	456,640	1,068,775	1,924,335
High quality reads	256,228	331,441	726,432	1,314,101
Total bases (bp)	100,671,040	139,089,398	300,240,405	540,000,843
Average read length (bp)	392.90	419.65	413.31	410.93

**Figure 2:**
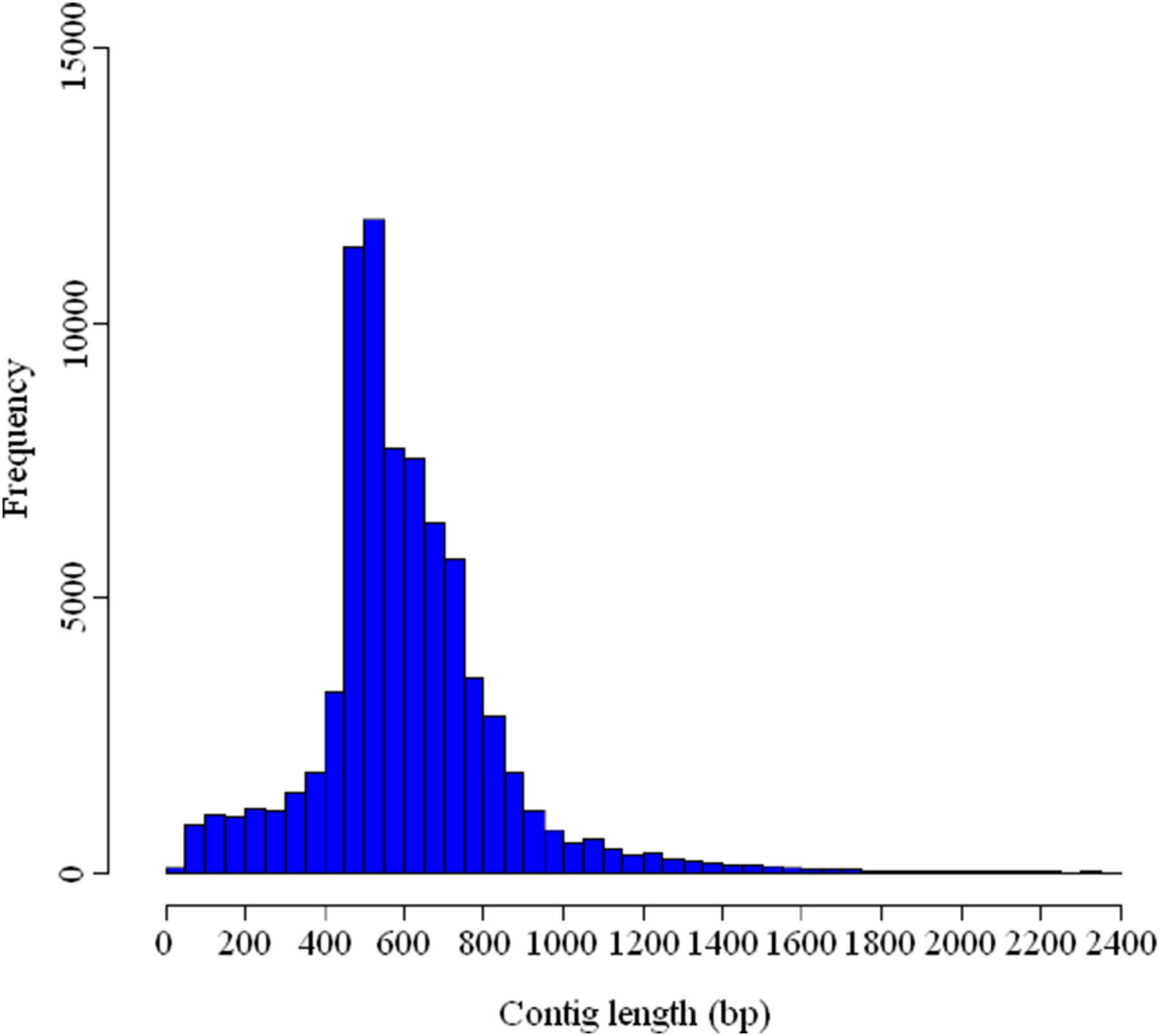
The size distribution of contigs resulted from de

De novo assembly produced 76,778 contigs, with 495,947 reads remaining as singletons (Table 2). Contigs consisted of 818,154 reads, representing approximately 46.5 Mbp data in size. The average length of contigs was 606 bp, and the N50 was 639 bp. A total of 4,058 contigs were longer than 1 kbp, with the longest one being 3,579 bp. The sequencing coverage of contigs ranged from 1 to 565, with an average of 5.4. The size distribution of contigs is shown in Figure 2. A significantly positive correlation (*r* = 0.355, *P* = 0.011) was found between the length of contig and the number of reads assembled. A regress formula was estimated as follows: *y* = 810+0.53*x* (*r^2^* = 0.126 and *P* = 0.011), where *y* represents the length of the contig, and *x* represents the number of reads assembled.

**Table 2:** Summary of contigs generated by de

Item	Number
Number of contigs	76,778
Total bases of contigs (bp)	46,525,023
Average length of contigs (bp)	605.97
Largest contig length (bp)	3,579
Number of contigs (≥1 kbp)	4058
Number of reads in contigs	818,154
N50 of contigs (bp)	639
Number of singletons	495,947

### Gene annotation

A total of 84,985 original unigenes and 78,268 final unigenes were identified respectively. Final unigenes consisted of 28,534 contigs and 49,734 singletons, of which, 6,414 showed significant BLASTX hits to known proteins in UniProt database, and 3,872 matched the known proteins in NR database. Moreover, 44,433 sequences were identified (E≤0.00001) from the original unigenes using BLASTN against the NCBI NT database. All final unigenes and their annotation information are listed in File S1 and File S2.

### GO analysis

**Figure 3:**
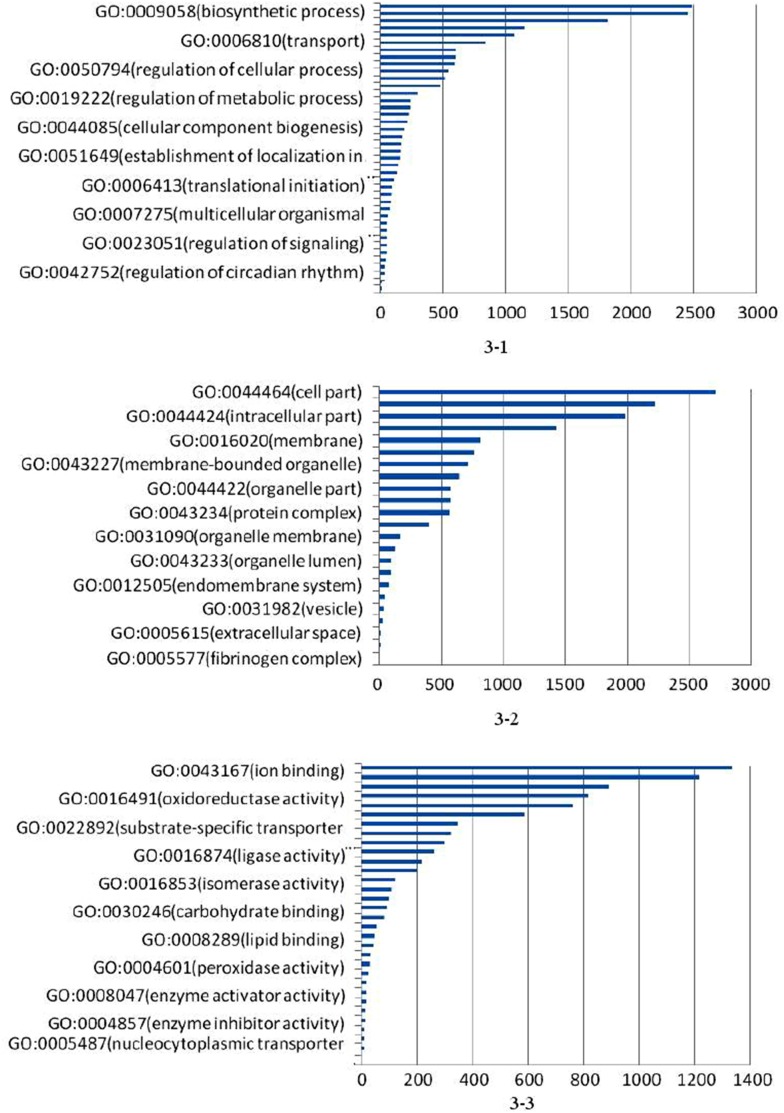
The classification of unigenes in three GO categories (level 3). Figure 3–1 indicated biological process; Figure 3–2 indicated cellular component; Figure 3–3 indicated molecular function; The x-axis indicated the number of unigenes in a process; The y-axis indicated GO process.

The annotated unigenes were classified into three GO categories: biological process, cellular component, and molecular function. Assignment results of unigenes in these three GO categories (level 3) are shown in Figure 3. For biological process, 3,908 unigenes were assigned to over 38 terms, of which biosynthetic process (GO: 0009058) and transport (GO: 0006810) were the dominant terms, followed by the regulation of cellular process (GO: 0050794). A total of 4,684 unigenes were classified into 30 terms in cellular component process, of which cell part (GO: 0044464) was the most represented term, followed by intracellular part (GO: 0044424) and membrane (GO: 0016020) terms. Meanwhile, 2,750 unigenes were distributed in 20 terms in molecular function process, of which ion binding (GO: 0043167) and oxidoreductase activity (GO: 0016491) terms were highly represented. From these categories, many growth-related and immune-related genes were found, such as beta-actin, growth hormone-inducible transmembrane protein, enoyl-CoA hydratase, serine protease inhibitor, transforming growth factor beta regulator, and transforming growth factor beta receptor (see File S2). In addition, a number of mitochondrial genes or partial gene fragments were detected that have been employed to obtain a complete mitochondrial genome of *S. paramamosain* [9].

### KEGG pathway analysis

**Figure 4:**
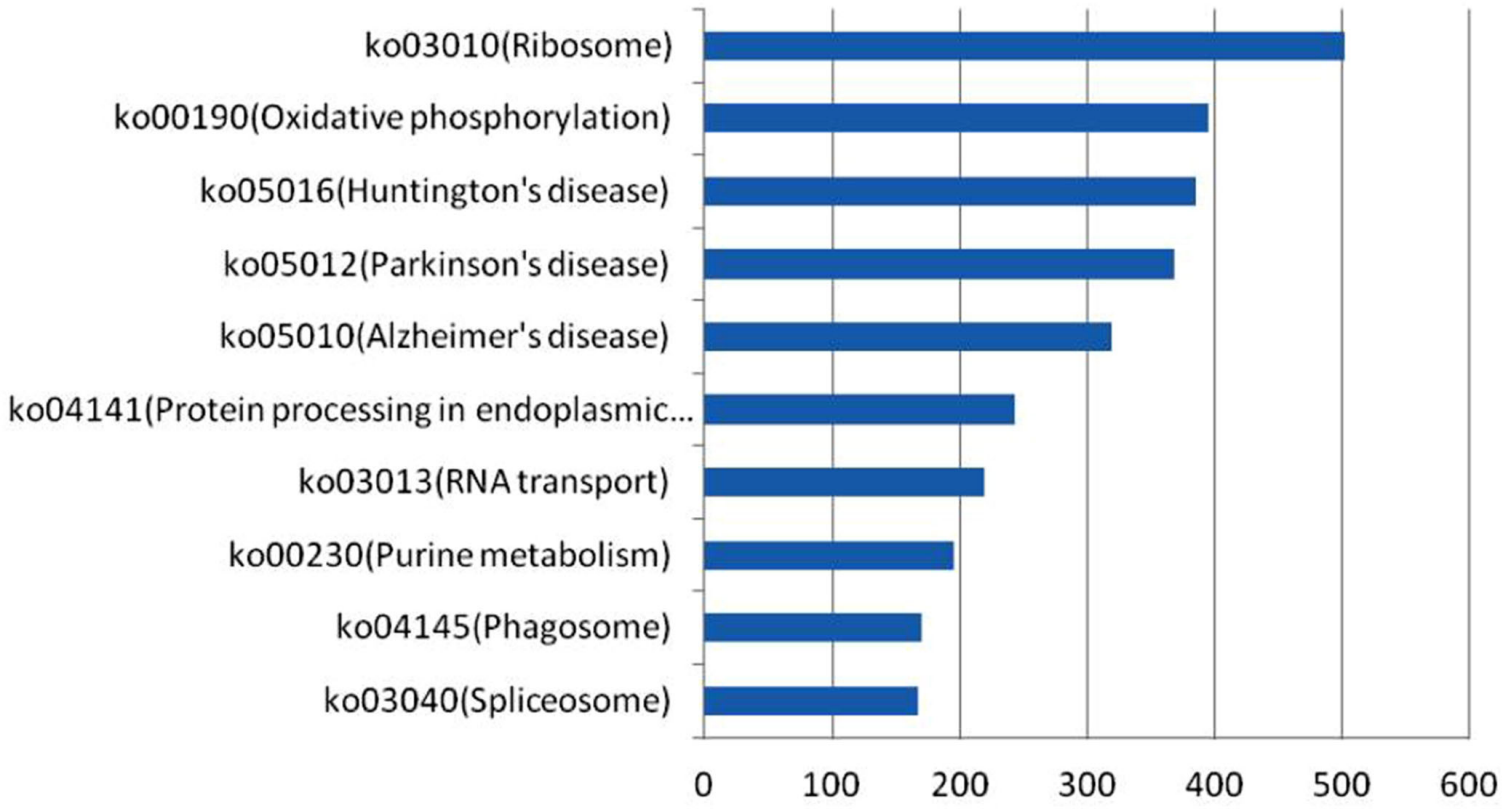
The ten most representive pathways resulted from KEGG pathway annotation. The x-axis indicated the number of unigenes in a pathway; The y-axis indicated the ten representive pathway.

In this study, 4,878 unigenes were classified into 281 KEGG pathways. Ribosome (KO: 03010) was the most dominant pathway with 505 unigenes, followed by oxidative phosphorylation (KO: 00190), Huntington’s disease (KO: 05016), Parkinson’s disease (KO: 05012), and Alzheimer’s disease (KO: 05010). These four pathways contained 391, 380, 371, and 320 unigenes, respectively. The rest of the pathways included relatively fewer unigenes, such as purine metabolism (KO: 00230), phagosome (KO: 04145), and spliceosome (KO: 03040). The 10 most represented pathways and their unigene numbers are shown in Figure 4. Furthermore, we found a few cases wherein multi-unigenes were annotated as the same expected gene that indicated that these unigenes were different fragments of a single transcript or different members of a gene family.

### Microsatellite and SNP discovery

A total of 19,011 microsatellites were detected from unigenes, with the repeat motifs ranging from 1 to 6 bp. Di-nucleotide repeats (*N* = 8161) were the most common microsatellites (42.9%), of which AC/GT repeats were the most represented (*N* = 5619), followed by CT/GA (*N* = 2211), AT (*N* = 316), and CG (*N* = 15). A large number of one nucleotide repeat motifs (*N* = 6636) were also found (34.9%), of which T/A repeats were the most abundant (*N* = 4293). A certain number of microsatellite pairs were found being very close to each other (less than 100 bp). The microsatellites identified from unigenes are listed in File S3.

Of 78 microsatellite loci, 30 (38.5%) showed polymorphism in the wild population. A total of 174 alleles were detected, with an average of 5.8 per locus. The observed heterozygosity (*H*_O_), expected heterozygosity (*H*_E_), and polymorphism information content per locus ranged from 0.13 to 1.00, 0.21 to 0.91, and 0.20 to 0.89, respectively. Two loci significantly deviated from Hardy–Weinberg equilibrium (*P*<0.01), and no evidence for stuttering and allelic dropout were found. Furthermore, no significant linkage disequilibrium between pairs of loci was found. Details about the 30 polymorphic microsatellites are listed in File S4.

Aside from microsatellites, a total of 37,063 potential SNPs and 57,220 indels were identified from 28,534 unigenes (coverage threshold≥5). The overall density of SNP, except indels, was one every 466 bp. Aside from bi-allelic SNPs, many tri- and even quad-allelic SNPs were found in the transcriptome. A large number of SNPs were detected in known-function genes, such as beta-actin, arginine kinase, and vasa-like protein that are useful for illustrating gene impact on economically important traits. Potential SNPs are shown in File S5.

## Discussion

Currently, an increasing number of organisms have been sequenced by next-generation high-throughput sequencing technique, which shows great potential for rapidly providing numerous genomic and genetic data [33]. This study first conducted high-throughput 454 sequencing of transcriptome in the mud crab. To obtain as many transcripts or ESTs as possible, especially low-expressed ESTs, we did not normalize the sequencing library. Normalized library is useful to reduce the over-representation of the most common transcripts, and it was constructed in pines (*Pinus* spp.) [23] and crucian carp (*Carassius auratus*) [34]. Non-normalized and normalized libraries were constructed in sea cucumber (*Apostichopus japonicus*) [35].

In this study, 540 Mbp data were generated in a total of one run reaction that consisted of 1,314,101 high quality reads. The amount of these reads were much more than that obtained from pines (*Pinus* spp., 586,732 reads in one run) [23], *Ascaris suum* (580,000 reads in one run) [36], and copepod (*Calanus sinicus*, 700,000 reads in one run) [37]. The average length of high quality reads was 411 bp, which was larger than those generated in above studies: 306 bp in pines, 356 bp in *A. suum,* and 355 bp in copepod. Further, the reads generated in our study were also larger than that produced in common carp (*Cyprinus carpio*, 321 bp) [38].

A total of 76,778 contigs were produced by de novo assembly. However, 495,947 reads failed to integrate into any contigs, which represented 37.7% of the total high quality reads. This percentage was higher than that reported in coral (*A. millepora*, 10%) [22] and copepod (*C. sinicus*, 16.5%) [37], but lower than that detected in pines (*Pinus* spp., 40.9%) [23]. The high proportion of singletons may be due to sequencing errors, limited sequencing coverage, low-level expressed genes, and the assembly algorithm.

To discover as many known genes as possible, two protein databases (UniProt and NR) were employed. As a result, a small number of unigenes were annotated, of which 6,414 (8.2%) and 3,872 (4.9%) were annotated in UniProt and NR databases, respectively. This result can be attributed to the existence of rare genome sequences of closely related organisms in public databases and the absence of whole genome sequences of crabs. The annotation rate in this study is much lower than that found in other aquatic animals, such as yesso scallop (*P. yessoensis*, 27.9%) [25], sea cucumber (*A. japonicus*, 39.1%) [35], and crucian carp (*C. auratus*, 17.4%) [34]. Besides, this annotation rate was also lower than that reported in the mud crab (*S. paramamosain*) in previous study, wherein 847 among 3,837 unique transcripts were annotated in NR database (the annotation rate was 22.1%) [20].

An important task of high-throughput sequencing is to identify the expression information of unigenes in biochemical process. In this study, a total of 3,908, 4,684, and 2,750 unigenes were assigned to biological process, cellular component, and molecular function, respectively. Among these unigenes, 4,878 were further classified into 281 KEGG pathways. The known genes identified in this study may have an important role in biochemical process including growth, development, and disease resistance. These findings will be very helpful for gene cloning and function analysis in *S. paramamosain*. Based on these unigenes, we have successfully achieved the complete cDNA sequence of beta-actin gene, and characterized its unstable expression profile among different tissues of *S. paramamosain* [39]. We also obtained the complete mitochondrial genome sequence and figured out the gene arrangement structure and phylogenetic relationship [9].

Compared with traditional methods, next-generation transcriptome sequencing technique has shown great potential in development of microsatellite and SNP markers. This technique has also been broadly applied in many aquatic organisms [34], [40]–[42]. In this study, several kinds of microsatellites were identified from transcriptome, of which AC/GT repeats were the most abundant. Meanwhile, a large number of one nucleotide repeat motifs were detected, however, this kind of repeat is not used for microsatellite amplification in practice. The polymorphism rate of the microsatellite loci was 38.5%. Based on this rate, we could predict in advance that approximate 7,319 polymorphic microsatellite markers can be developed from this transcriptome data set. The genetic diversity level of these 30 polymorphic microsatellites (average *N*_a_ and *H*_O_ were 5.8 and 0.72, respectively) was slightly lower than that of the genome randomly derived microsatellites (average *N*_a_ and *H*_O_ were 6.8 and 0.76, respectively) [17] and nearly equal to that of gene-derived microsatellites (average *N*_a_ and *H*_O_ were 5.9 and 0.67, respectively) [43].

## Conclusions

We first conducted a 454 high-throughput sequencing on the mud crab (*Scylla paramamosain*) transcriptome that produced a total of 1,314,101 high quality reads, 78,268 unigenes, 19,011 microsatellites, and 37,063 potential SNPs. We then reported the development of 30 polymorphic microsatellites and genetic diversity of a wild population. These findings significantly enhanced our understanding of the genome structure and function of *S. paramamosain*, and they can facilitate further studies on genomics, and molecular breeding in crustaceans, especially in genus *Scylla* spp.

## Supporting Information

File S1 **Sequences of unigenes.**
(RAR)

File S2 **Unigene annotation, GO and KEGG information.**
(XLSX)

File S3 **Microsatellites detected in unigenes.**
(XLSX)

File S4 **Characterization of 30 polymorphic microsatellite markers derived from transcriptome sequences in S. paramamosain.**
(DOCX)

File S5 **Potential SNPs identified in unigenes.**
(XLSX)
