# Supplementary material for: Transcriptome Analysis of the Mud Crab (Scylla paramamosain) by 454 Deep Sequencing: Assembly, Annotation, and Marker Discovery
Source: PLoS One. 2014 Jul 23;9(7):e102668. doi: 10.1371/journal.pone.0102668 (PMC4108364; doi:10.1371/journal.pone.0102668)
Supplement: File S4 — Characterization of 30 polymorphic microsatellite markers derived from transcriptome sequences in S. paramamosain. (DOCX) [file pone.0102668.s004.docx]

Characterization of 30 polymorphic microsatellite markers derived from transcriptome sequences in *S. paramamosain*

| Locus | Repeat sequence | Primer sequence ( 5’ - 3’) | *T*_a_ (°C) | *N*_a_ /*N*_e_ | *H*_O_ | *H*_E_ | PIC | *P* | GenBank accession no. |
| --- | --- | --- | --- | --- | --- | --- | --- | --- | --- |
| Scpa80 | (ACC)_9_ | GGAGAAGTCCCGCATCGT  CCCGCTACCTCTACTCAACC | 59 | 4/3.7 | 1.00 | 0.74 | 0.68 | 0.058 | KF452324 |
| Scpa81 | (TG)_16_ | GCAATTCGGTATTATTTATC  AATCTTGTCGCACATCAC | 50 | 5/3.7 | 0.93 | 0.74 | 0.68 | 0.180 | KF452325 |
| Scpa82 | (GAG)_8_ | GGATGATATGGAAGCTGAGG  GATCTGTCCCGCTTACCA | 54 | 5/3.4 | 0.66 | 0.72 | 0.65 | 0.383 | KF452326 |
| Scpa83 | (TAC)_15_ | CAGGGAAGACGATAGATGC  TGGTTGAAAGGATGGGTA | 52 | 7/4.8 | 0.90 | 0.81 | 0.77 | 0.571 | KF452327 |
| Scpa84 | (CA)_12_ | CACAGTTGTGGCACCCTA  GTATTTGTAAACCTTTCAGTGTC | 54 | 4/3.3 | 0.72 | 0.71 | 0.64 | 0.409 | KF452328 |
| Scpa85 | (GT)_13_ | GTGAGGAAAGCAAAGAACA  CAGTTTCATCATCAGCCAT | 51 | 7/5.2 | 0.71 | 0.82 | 0.78 | 0.677 | KF452329 |
| Scpa86 | (TAT)_15_ | GTGGCTTTAGTGGGTAGA  GCTGTGAGACCTCAAGATAT | 53 | 9/5.6 | 0.83 | 0.84 | 0.80 | 0.767 | KF452330 |
| Scpa87 | (GT)_11_ | GCCACTGAAGCTTTTAATC  CTCCTCCTATCTACACTACTTG | 53 | 6/4.6 | 0.89 | 0.80 | 0.75 | 0.171 | KF452331 |
| Scpa89 | (GT)_13_ | GCTGAGGAGGCTGACTTG  TCACCCACGGTTACATTTG | 55 | 5/3.1 | 0.55 | 0.81 | 0.62 | 0.814 | KF452333 |
| Scpa90 | (CT)_9_ | CTCACCAGCATGAACATC  CTCGCTTTACGCTTAGAC | 53 | 7/5.4 | 0.87 | 0.83 | 0.79 | 0.615 | KF452334 |
| Scpa91 | (CA)_14_ | CCACCGTCACATCAGGAAG  AGATGGCAGCAAGCAAGAC | 57 | 6/3.5 | 0.61 | 0.72 | 0.66 | 0.794 | KF452335 |
| Scpa92 | (CAGC)_6_(CAGT)_10_ | GAGGGAAGTAACTGGAAGA  GTTGCTAAATAATGCTGGA | 53 | 9/5.4 | 0.91 | 0.83 | 0.79 | 0.331 | KF452336 |
| Scpa94 | (CA)_8_ | TGTAATCAGAGGCAGCAGT  AACGACCACAAATAAGGAAC | 53 | 7/5.8 | 0.90 | 0.84 | 0.81 | 0.714 | KF452338 |
| Scpa95 | (GT)_13_ | GTCCCATTTGCCTCACTG  TGTATGTGCGTGCATGTTTA | 53 | 4/2.9 | 0.60 | 0.67 | 0.58 | 0.247 | KF452339 |
| Scpa97 | (TA)_8_ | ATGTTGCCCCTTTACCTC  AGAAAATGCGAACCTTGA | 48 | 4/3.6 | 0.83 | 0.73 | 0.67 | 0.453 | KF452341 |
| Scpa98 | (AC)_11_A(AC)_7_ | CGAGCAACTTGATGGGACA  GGCTGGAAGCAGGCTAAAT | 56 | 3/2.1 | 0.50 | 0.53 | 0.46 | 0.792 | KF452342 |
| Scpa99 | (AC)_15_ | TAAATGCGGCACAAAGCG  CCAGGATGACCAACAAAGTAAAT | 51 | 7/5.0 | 0.55 | 0.81 | 0.77 | 0.003 ^*^ | KF452343 |
| Scpa100 | (GT)_13_ | CAGCCCAAGTGAGATACC  ATTGAATCTCTGGGTGACTT | 50 | 5/2.5 | 0.71 | 0.61 | 0.52 | 0.644 | KF452344 |
| Scpa101 | (CCTGT)_8_ | GTGTGGGAGTTTGTCACTTATT  AGGTAAGTGTAAGGTGGGATT | 51 | 12/9.9 | 0.86 | 0.91 | 0.89 | 0.543 | KF452345 |
| Scpa102 | (AGT)_14_N_36_(GTA)_9_ | CGCCTCATCATCTCACTG  TATATAGAAGCCCTACTCCCT | 54 | 8/5.8 | 0.94 | 0.84 | 0.80 | 0.681 | KF452346 |
| Scpa104 | (CA)_10_ | GGCGACCGACTACACTTTG  AGTGGAGGAGGTTGAGGGA | 57 | 6/4.4 | 0.87 | 0.79 | 0.74 | 0.226 | KF452348 |
| Scpa105 | (CA)_8_ | TGTCGAGGGTAAAGGAGG  CGTGGCAAAGATTGGTAA | 49 | 4/1.9 | 0.59 | 0.49 | 0.44 | 0.758 | KF452349 |
| Scpa108 | (CAC)_8_ | GAAAAAGCCGGAAGACAAG  GGAACCGAAACCTGAACC | 54 | 4/2.9 | 0.63 | 0.66 | 0.58 | 0.526 | KF452352 |
| Scpa109 | (CA)_8_ | GTTTGTCCTACTGGATATTTCACC  GACGAGAGACAATGGTTTAGTGAG | 57 | 7/2.0 | 0.59 | 0.51 | 0.49 | 0.977 | KF452353 |
| Scpa110 | (AC)_12_ | GCAGAGTGGCTATTACGG  CCTTGTCTTTTCATGTCCAT | 52 | 4/3.4 | 0.86 | 0.72 | 0.65 | 0.225 | KF452354 |
| Scpa112 | (AAC)_8_ | CAATAAGTCGGTAGTTAGTAAGAAT  GACAACGACTGACGACTACTG | 57 | 5/1.3 | 0.13 | 0.21 | 0.20 | 0.000 ^*^ | KF452356 |
| Scpa113 | (TG)_15_ | GGAGGAACTGGGTTGTGA  AATGCTGCTGTACGTGGC | 55 | 4/2.0 | 0.27 | 0.50 | 0.40 | 0.125 | KF452357 |
| Scpa114 | (GT)_15_ | GGATTGCTTCAGGGTCTACTAC  GGTGTAGAGGCAGAGCGTTA | 57 | 6/4.0 | 0.91 | 0.76 | 0.71 | 0.249 | KF452358 |
| Scpa115 | (ACT)_14_ | GGTGGCACTTCAACTTCTG  TCTCCCACCCTCACAGAG | 57 | 7/3.3 | 0.67 | 0.70 | 0.66 | 0.592 | KF452359 |
| Scpa116 | (TG)_8_ | GTATCAACGCAGAGTGGC  TCCTTGAATCCAAAACCA | 52 | 3/1.8 | 0.58 | 0.46 | 0.40 | 0.183 | KF452360 |
| Mean | / | / | / | 5.8/3.9 | 0.72 | 0.70 | 0.65 | / | / |

*T*_a_, annealing temperature; *N*_a_, observed number of alleles; *N*_e_, effective number of alleles; *H*_O_, observed heterozygosity; *H*_E_, expected heterozygosity; PIC, polymorphism information content; *P*, Chi-square tests for Hardy-Weinberg equilibrium (HWE); ^*^, significant (*P* value < 0.01).
